# Supplementary material for: Treatment resistant depression in electronic health records: definitions matter
Source: BMC Psychiatry. 2026 Apr 24;26:453. doi: 10.1186/s12888-026-08085-y (PMC13245096; doi:10.1186/s12888-026-08085-y)
Supplement: Supplementary file 1 — Supplementary Material 1 [file 12888_2026_8085_MOESM1_ESM.docx]

**Table S1.** Number of individuals removed during data cleaning due to the exclusion criteria of other psychiatric diagnoses. Note that diagnoses are not mutually exclusive.

|  | **DataLoch**  (N excluded = 26,949) | **UK Biobank**  (N excluded = 3,671) | **Generation Scotland**  (N excluded = 196) |
| --- | --- | --- | --- |
| **Drug misuse** | 10,085 | 628 | 80 |
| **Alcohol misuse** | 21,417 | 2,672 | 122 |
| **Bipolar disorder** | 1,358 | 617 | 40 |
| **Psychosis** | 1,912 | 514 | 28 |

**Table S2.** The proportion of individuals included in each TRD definition, as a proportion of those in the analytic samples, for the Dataloch cohort. Rows indicate the number of antidepressant switches required to be included in the definition, columns indicate whether the definition is based only on item switches (lower is more inclusive), or whether individuals can also enter the definition by experiencing one or more augmentation or one or more switch of antidepressant class (more conditions is more inclusive).

|  | **Switch-only** | **Switches or Augmentation** | **Switches or Augmentation or Class switch** |
| --- | --- | --- | --- |
| **1+ Switch** | 27% | 27% | 27% |
| **2+ Switches** | 10% | 11% | 23% |
| **3+ Switches** | 4% | 4% | 23% |

**Table S3.** The proportion of individuals included in each TRD definition, as a proportion of those in the analytic samples, for the UK Biobank cohort. Rows indicate the number of antidepressant switches required to be included in the definition, columns indicate whether the definition is based only on item switches (lower is more inclusive), or whether individuals can also enter the definition by experiencing one or more augmentation or one or more switch of antidepressant class (more conditions is more inclusive).

|  | **Switch-only** | **Switches or Augmentation** | **Switches or Augmentation or Class switch** |
| --- | --- | --- | --- |
| **1+ Switch** | 4% | 4% | 4% |
| **2+ Switches** | 2% | 2% | 3% |
| **3+ Switches** | 1% | 1% | 3% |

**Table S4.** The proportion of individuals included in each TRD definition, as a proportion of those in the analytic samples, for the Generation Scotland cohort. Rows indicate the number of antidepressant switches required to be included in the definition, columns indicate whether the definition is based only on item switches (lower is more inclusive), or whether individuals can also enter the definition by experiencing one or more augmentation or one or more switch of antidepressant class (more conditions is more inclusive).

|  | **Switch-only** | **Switches or Augmentation** | **Switches or Augmentation or Class switch** |
| --- | --- | --- | --- |
| **1+ Switch** | 20% | 21% | 21% |
| **2+ Switches** | 4% | 4% | 12% |
| **3+ Switches** | 1% | 2% | 12% |

**Table S5.** Descriptive statistics for individuals included in each definition, DataLoch cohort (N = 51,283). P-values relate to Kruskal-Wallis tests (for numeric variables) and Chi-Square tests (for categorical variables) of between-definition differences.

|  | **1+ switch (N=13,643)** | **1+ switch**  **Augmentation (N=13,729)** | **1+ switch**  **Augmentation + 1+ between-class**  **(N=13,729)** | **2+ switch (N=5,331)** | **2+ switch**  **Augmentation (N=5,495)** | **2+ switch**  **Augmentation**  **1+ between-class**  **(N=11,972)** | **3+ switch (N=2,053)** | **3+ switch**  **Augmentation (N=2,279)** | **3+ switch**  **Augmentation**  **1+ between-class**  **(N=11,811)** | **p-value** |
| --- | --- | --- | --- | --- | --- | --- | --- | --- | --- | --- |
| Age at end of follow-up (years) |  |  |  |  |  |  |  |  |  | <0.001 |
| Median  (Q1, Q3) | 63.00  (55.00, 76.00) | 63.00  (55.00, 76.00) | 63.00  (55.00, 76.00) | 62.00  (54.00, 74.00) | 62.00  (54.00, 74.00) | 64.00  (55.00, 77.00) | 61.00  (54.00, 71.00) | 61.00  (54.00, 72.00) | 64.00  (55.00, 77.00) |  |
| Sex |  |  |  |  |  |  |  |  |  | 0.021 |
| Female | 10044 (73.6%) | 10103 (73.6%) | 10103 (73.6%) | 3990 (74.8%) | 4102 (74.6%) | 8835 (73.8%) | 1572 (76.6%) | 1728 (75.8%) | 8714 (73.8%) |  |
| Male | 3599 (26.4%) | 3626 (26.4%) | 3626 (26.4%) | 1341 (25.2%) | 1393 (25.4%) | 3137 (26.2%) | 481 (23.4%) | 551 (24.2%) | 3097 (26.2%) |  |
| SIMD Quintile |  |  |  |  |  |  |  |  |  | <0.001 |
| Median  (Q1, Q3) | 3.00  (2.00, 4.00) | 3.00  (2.00, 4.00) | 3.00  (2.00, 4.00) | 3.00  (2.00, 4.00) | 3.00  (2.00, 4.00) | 3.00  (2.00, 4.00) | 3.00  (2.00, 4.00) | 3.00  (2.00, 4.00) | 3.00  (2.00, 4.00) |  |
| Ethnicity |  |  |  |  |  |  |  |  |  | 0.995 |
| Asian | 194 (1.4%) | 196 (1.4%) | 196 (1.4%) | 70 (1.3%) | 72 (1.3%) | 176 (1.5%) | 24 (1.2%) | 26 (1.1%) | 175 (1.5%) |  |
| Mixed | 45 (0.3%) | 46 (0.3%) | 46 (0.3%) | 21 (0.4%) | 22 (0.4%) | 39 (0.3%) | 10 (0.5%) | 11 (0.5%) | 38 (0.3%) |  |
| Not Stated | 934 (6.8%) | 943 (6.9%) | 943 (6.9%) | 332 (6.2%) | 347 (6.3%) | 814 (6.8%) | 127 (6.2%) | 143 (6.3%) | 802 (6.8%) |  |
| Other | 158 (1.2%) | 158 (1.2%) | 158 (1.2%) | 60 (1.1%) | 60 (1.1%) | 142 (1.2%) | 23 (1.1%) | 23 (1.0%) | 141 (1.2%) |  |
| White | 12312 (90.2%) | 12386 (90.2%) | 12386 (90.2%) | 4848 (90.9%) | 4994 (90.9%) | 10801 (90.2%) | 1869 (91.0%) | 2076 (91.1%) | 10655 (90.2%) |  |
| Number of MDD codes in EHRs |  |  |  |  |  |  |  |  |  | <0.001 |
| Median  (Q1, Q3) | 3.00  (2.00, 6.00) | 3.00  (2.00, 6.00) | 3.00  (2.00, 6.00) | 4.00  (2.00, 7.00) | 4.00  (2.00, 7.00) | 3.00  (2.00, 6.00) | 4.00  (2.00, 8.00) | 4.00  (2.00, 8.00) | 3.00  (2.00, 6.00) |  |
| Treatment time (months) |  |  |  |  |  |  |  |  |  | <0.001 |
| Median  (Q1, Q3) | 16.00  (5.03, 43.99) | 16.03  (5.03, 43.99) | 16.03  (5.03, 43.99) | 17.02  (5.98, 44.97) | 17.97  (5.98, 45.99) | 16.03  (5.03, 44.97) | 16.03  (5.98, 42.97) | 18.04  (6.05, 48.00) | 16.03  (5.03, 43.99) |  |
| Age of first MDD code (years) |  |  |  |  |  |  |  |  |  | <0.001 |
| Median  (Q1, Q3) | 45.00  (35.00, 58.00) | 45.00  (35.00, 58.00) | 45.00  (35.00, 58.00) | 43.00 (34.00, 55.00) | 43.00  (35.00, 55.00) | 45.00  (36.00, 59.00) | 41.00  (34.00, 53.00) | 42.00  (34.00, 53.00) | 46.00  (36.00, 59.00) |  |
| Destination antidepressant class |  |  |  |  |  |  |  |  |  | <0.001 |
| MAOIs | 29 (0.2%) | 29 (0.2%) | 29 (0.2%) | 24 (0.5%) | 24 (0.4%) | 27 (0.2%) | 15 (0.7%) | 15 (0.7%) | 28 (0.2%) |  |
| Other | 2179 (16.0%) | 2192 (16.0%) | 2192 (16.0%) | 859 (16.1%) | 893 (16.3%) | 2203 (18.4%) | 295 (14.4%) | 341 (15.0%) | 2224 (18.8%) |  |
| SARIs | 1039 (7.6%) | 1046 (7.6%) | 1046 (7.6%) | 458 (8.6%) | 466 (8.5%) | 1077 (9.0%) | 186 (9.1%) | 196 (8.6%) | 1075 (9.1%) |  |
| SNRIs | 2307 (16.9%) | 2321 (16.9%) | 2321 (16.9%) | 1181 (22.2%) | 1215 (22.1%) | 2376 (19.8%) | 510 (24.8%) | 560 (24.6%) | 2434 (20.6%) |  |
| SSRIs | 4776 (35.0%) | 4817 (35.1%) | 4817 (35.1%) | 1564 (29.3%) | 1629 (29.6%) | 3156 (26.4%) | 575 (28.0%) | 660 (29.0%) | 2958 (25.0%) |  |
| TCAs | 3313 (24.3%) | 3324 (24.2%) | 3324 (24.2%) | 1245 (23.4%) | 1268 (23.1%) | 3133 (26.2%) | 472 (23.0%) | 507 (22.2%) | 3092 (26.2%) |  |

**Table S6.** Descriptive statistics for individuals included in each definition, UK Biobank cohort (N = 19,641). P-values relate to Kruskal-Wallis tests (for numeric variables) and Chi-Square tests (for categorical variables) of between-definition differences.

|  | **1+ switch**  **(N=777)** | **1+ switch Augmentation**  **(N=785)** | **1+ switch Augmentation**  **1+ between-class**  **(N=785)** | **2+ switch**  **(N=475)** | **2+ switch Augmentation**  **(N=485)** | **2+ switch Augmentation**  **1+ between-class**  **(N=684)** | **3+ switch**  **(N=248)** | **3+ switch Augmentation**  **(N=260)** | **3+ switch Augmentation**  **1+ between-class**  **(N=654)** | **p-value** |
| --- | --- | --- | --- | --- | --- | --- | --- | --- | --- | --- |
| Age^1^ |  |  |  |  |  |  |  |  |  | 0.988 |
| Median  (Q1, Q3) | 56.00  (48.00, 62.00) | 56.00  (48.00, 62.00) | 56.00  (48.00, 62.00) | 56.00  (48.00, 62.00) | 56.00  (48.00, 62.00) | 56.00  (49.00, 62.00) | 56.50  (49.00, 62.00) | 57.00  (49.00, 62.00) | 57.00  (49.00, 62.00) |  |
| Sex^1^ |  |  |  |  |  |  |  |  |  | 0.455 |
| Female | 586 (75.4%) | 591 (75.3%) | 591 (75.3%) | 375 (78.9%) | 381 (78.6%) | 522 (76.3%) | 199 (80.2%) | 207 (79.6%) | 502 (76.8%) |  |
| Male | 191 (24.6%) | 194 (24.7%) | 194 (24.7%) | 100 (21.1%) | 104 (21.4%) | 162 (23.7%) | 49 (19.8%) | 53 (20.4%) | 152 (23.2%) |  |
| Townsend Deprivation^1^ |  |  |  |  |  |  |  |  |  | 0.755 |
| N-Miss | 1 | 1 | 1 | 1 | 1 | 1 | 0 | 0 | 1 |  |
| Median  (Q1, Q3) | -0.14  (-2.74, 3.71) | -0.14  (-2.74, 3.69) | -0.14  (-2.74, 3.69) | 0.04  (-2.58, 4.26) | 0.02  (-2.60, 4.17) | -0.10  (-2.74, 3.65) | 0.16  (-2.78, 4.42) | 0.16  (-2.78, 4.41) | -0.10  (-2.74, 3.59) |  |
| Educational qualification |  |  |  |  |  |  |  |  |  | 0.999 |
| N-Miss | 1 | 1 | 1 | 1 | 1 | 1 | 0 | 0 | 1 |  |
| Primary | 218 (28.1%) | 219 (27.9%) | 219 (27.9%) | 143 (30.2%) | 145 (30.0%) | 195 (28.6%) | 87 (35.1%) | 91 (35.0%) | 186 (28.5%) |  |
| Lower Secondary | 163 (21.0%) | 165 (21.0%) | 165 (21.0%) | 96 (20.3%) | 98 (20.2%) | 140 (20.5%) | 52 (21.0%) | 54 (20.8%) | 132 (20.2%) |  |
| Upper Secondary | 214 (26.5%) | 218 (26.7%) | 218 (26.7%) | 122 (24.5%) | 127 (24.9%) | 187 (26.2%) | 57 (21.6%) | 62 (22.2%) | 178 (26.1%) |  |
| Post-Secondary | 91 (11.7%) | 92 (11.7%) | 92 (11.7%) | 65 (13.7%) | 66 (13.6%) | 84 (12.3%) | 28 (11.3%) | 29 (11.2%) | 79 (12.1%) |  |
| Degree | 208 (26.8%) | 212 (27.0%) | 212 (27.0%) | 117 (24.7%) | 122 (25.2%) | 181 (26.5%) | 55 (22.2%) | 60 (23.1%) | 174 (26.6%) |  |
| Unknown | 15 (1.9%) | 15 (1.9%) | 15 (1.9%) | 8 (1.7%) | 8 (1.7%) | 15 (2.2%) | 6 (2.4%) | 6 (2.3%) | 15 (2.3%) |  |
| Ethnicity^1^ |  |  |  |  |  |  |  |  |  | 1.000 |
| N-Miss | 3 | 3 | 3 | 3 | 3 | 3 | 1 | 1 | 2 |  |
| Asian | 9 (1.2%) | 9 (1.2%) | 9 (1.2%) | 6 (1.3%) | 6 (1.2%) | 7 (1.0%) | 4 (1.6%) | 4 (1.5%) | 7 (1.1%) |  |
| Mixed | 3 (0.4%) | 3 (0.4%) | 3 (0.4%) | 1 (0.2%) | 1 (0.2%) | 3 (0.4%) | 0 (0.0%) | 0 (0.0%) | 3 (0.5%) |  |
| Not Stated | 2 (0.3%) | 2 (0.3%) | 2 (0.3%) | 1 (0.2%) | 2 (0.4%) | 2 (0.3%) | 1 (0.4%) | 2 (0.8%) | 2 (0.3%) |  |
| Other | 9 (1.2%) | 9 (1.2%) | 9 (1.2%) | 7 (1.5%) | 7 (1.5%) | 8 (1.2%) | 3 (1.2%) | 3 (1.2%) | 8 (1.2%) |  |
| White | 751 (97.0%) | 759 (97.1%) | 759 (97.1%) | 457 (96.8%) | 466 (96.7%) | 661 (97.1%) | 239 (96.8%) | 250 (96.5%) | 632 (96.9%) |  |
| Number of MDD codes in EHRs |  |  |  |  |  |  |  |  |  | 0.958 |
| Median  (Q1, Q3) | 1.00 (1.00, 3.00) | 1.00  (1.00, 3.00) | 1.00  (1.00, 3.00) | 2.00  (1.00, 3.00) | 2.00  (1.00, 3.00) | 1.00  (1.00, 3.00) | 2.00  (1.00, 3.00) | 2.00  (1.00, 3.00) | 1.00  (1.00, 3.00) |  |
| Treatment time (months) |  |  |  |  |  |  |  |  |  | 0.522 |
| Median  (Q1, Q3) | 5.91  (0.00, 27.10) | 6.08  (0.00, 27.43) | 6.08  (0.00, 27.43) | 5.98  (0.00, 27.61) | 6.34  (0.00, 29.70) | 5.39  (0.00, 23.80) | 5.63  (0.00, 27.34) | 6.47  (0.00, 32.00) | 4.44  (0.00, 22.31) |  |
| MDD Polygenic Risk Score |  |  |  |  |  |  |  |  |  | 1.000 |
| N-Miss | 27 | 28 | 28 | 21 | 22 | 27 | 9 | 10 | 25 |  |
| Median (Q1, Q3) | 0.00  (0.00, 0.00) | 0.00  (0.00, 0.00) | 0.00  (0.00, 0.00) | 0.00  (0.00, 0.00) | 0.00  (0.00, 0.00) | 0.00  (0.00) | 0.00 (0.00) | 0.00  (0.00) | 0.00  (0.00) |  |
| Self-reported depression^2^ |  |  |  |  |  |  |  |  |  | 0.715 |
| N-Miss | 1 | 1 | 1 | 1 | 1 | 1 | 0 | 0 | 1 |  |
| No | 584 (75.3%) | 588 (75.0%) | 588 (75.0%) | 344 (72.6%) | 348 (71.9%) | 513 (75.1%) | 179 (72.2%) | 185 (71.2%) | 491 (75.2%) |  |
| Yes | 192 (24.7%) | 196 (25.0%) | 196 (25.0%) | 130 (27.4%) | 136 (28.1%) | 170 (24.9%) | 69 (27.8%) | 75 (28.8%) | 162 (24.8%) |  |
| Self-reported age of first diagnosis^3^ |  |  |  |  |  |  |  |  |  | 0.913 |
| N-Miss | 585 | 589 | 589 | 345 | 349 | 514 | 179 | 185 | 492 |  |
| Median  (Q1, Q3) | 45.52  (35.40, 53.74) | 45.25  (35.23, 53.74) | 45.25  (35.23, 53.74) | 44.54  (33.47, 53.11) | 44.48  (32.43, 52.90) | 45.25  (34.81, 53.99) | 44.46  (30.50, 51.59) | 44.28  (30.39, 51.54) | 44.92  (34.72, 53.99) |  |
| Family history of severe depression^2^ |  |  |  |  |  |  |  |  |  | 0.989 |
| N-Miss | 121 | 122 | 122 | 84 | 86 | 114 | 42 | 45 | 110 |  |
| No | 497 (75.8%) | 501 (75.6%) | 501 (75.6%) | 295 (75.4%) | 300 (75.2%) | 434 (76.1%) | 151 (73.3%) | 157 (73.0%) | 416 (76.5%) |  |
| Yes | 159 (24.2%) | 162 (24.4%) | 162 (24.4%) | 96 (24.6%) | 99 (24.8%) | 136 (23.9%) | 55 (26.7%) | 58 (27.0%) | 128 (23.5%) |  |
| Self-reported self harm^3^ |  |  |  |  |  |  |  |  |  | 1 |
| N-Miss | 603 | 611 | 611 | 374 | 384 | 537 | 200 | 212 | 514 |  |
| No | 163 (93.7%) | 163 (93.7%) | 163 (93.7%) | 94 (93.1%) | 94 (93.1%) | 137 (93.2%) | 45 (93.8%) | 45 (93.8%) | 130 (92.9%) |  |
| Yes | 11 (6.3%) | 11 (6.3%) | 11 (6.3%) | 7 (6.9%) | 7 (6.9%) | 10 (6.8%) | 3 (6.2%) | 3 (6.2%) | 10 (7.1%) |  |
| PHQ4 score^3^ |  |  |  |  |  |  |  |  |  | <0.001 |
| N-Miss | 17 | 17 | 17 | 12 | 12 | 15 | 7 | 7 | 15 |  |
| Median  (Q1, Q3) | 3.00  (1.00, 5.00) | 3.00  (1.00, 5.00) | 3.00  (1.00, 5.00) | 4.00  (1.00, 5.00) | 4.00  (1.00, 5.00) | 3.00  (1.00, 5.00) | 4.00  (2.00, 6.00) | 4.00  (1.00, 6.00) | 3.00  (1.00, 5.00) |  |
| CIDI severity^2^ |  |  |  |  |  |  |  |  |  | 0.805 |
| N-Miss | 602 | 610 | 610 | 373 | 383 | 536 | 199 | 211 | 513 |  |
| Median  (Q1, Q3) | 6.00  (5.00, 7.50) | 6.00  (5.00, 7.50) | 6.00  (5.00, 7.50) | 6.00  (5.00, 8.00) | 6.00  (5.00, 8.00) | 6.00  (5.00, 7.00) | 7.00  (5.00, 8.00) | 7.00  (5.00, 8.00) | 6.00  (5.00, 7.00) |  |
| Destination antidepressant class |  |  |  |  |  |  |  |  |  | 0.692 |
| MAOIs | 4 (0.5%) | 4 (0.5%) | 4 (0.5%) | 3 (0.6%) | 3 (0.6%) | 4 (0.6%) | 3 (1.2%) | 3 (1.2%) | 4 (0.6%) |  |
| Other | 78 (10.0%) | 79 (10.1%) | 79 (10.1%) | 58 (12.2%) | 60 (12.4%) | 79 (11.5%) | 35 (14.1%) | 37 (14.2%) | 82 (12.5%) |  |
| SARIs | 16 (2.1%) | 16 (2.0%) | 16 (2.0%) | 12 (2.5%) | 12 (2.5%) | 16 (2.3%) | 7 (2.8%) | 7 (2.8%) | 19 (2.9%) |  |
| SNRIs | 66 (8.5%) | 68 (8.7%) | 68 (8.7%) | 47 (9.9%) | 49 (10.1%) | 70 (10.2%) | 23 (9.3%) | 25 (9.6%) | 63 (9.6%) |  |
| SSRIs | 345 (44.4%) | 346 (44.1%) | 346 (44.1%) | 194 (40.8%) | 195 (40.2%) | 258 (37.7%) | 96 (38.7%) | 99 (38.1%) | 230 (35.2%) |  |
| TCAs | 268 (34.5%) | 272 (34.6%) | 272 (34.6%) | 161 (33.9%) | 166 (34.2%) | 257 (37.6%) | 84 (33.9%) | 89 (34.2%) | 256 (39.1%) |  |
| Any antidepressant helped^3^ |  |  |  |  |  |  |  |  |  | 1 |
| N-Miss | 664 | 651 | 651 | 398 | 406 | 577 | 210 | 220 | 551 |  |
| No | 18 (13.5%) | 18 (13.4%) | 18 (13.4%) | 10 (13.0%) | 10 (12.7%) | 13 (12.1%) | 5 (11.9%) | 5 (11.1%) | 13 (12.1%) |  |
| Yes | 115 (86.5%) | 116 (86.6%) | 116 (86.6%) | 67 (87.0%) | 69 (87.3%) | 94 (87.9%) | 33 (86.8%) | 35 (87.5%) | 88 (87.1%) |  |
| Non-drug treatment helped^3^ |  |  |  |  |  |  |  |  |  | 0.989 |
| N-Miss | 665 | 672 | 672 | 418 | 426 | 594 | 218 | 228 | 568 |  |
| No | 15 (12.7%) | 15 (12.6%) | 15 (12.6%) | 11 (18.3%) | 11 (17.5%) | 14 (14.9%) | 6 (18.2%) | 6 (16.7%) | 12 (13.3%) |  |
| Yes, at least a little | 103 (87.3%) | 104 (87.4%) | 104 (87.4%) | 49 (81.7%) | 52 (82.5%) | 80 (85.1%) | 27 (81.8%) | 30 (83.3%) | 78 (86.7%) |  |
| GAD7 severity^3^ |  |  |  |  |  |  |  |  |  | 0.130 |
| N-Miss | 602 | 610 | 610 | 373 | 383 | 536 | 199 | 211 | 513 |  |
| Median  (Q1, Q3) | 2.00  (0.00, 6.00) | 2.00  (0.00, 6.00) | 2.00  (0.00, 6.00) | 4.00  (0.00, 6.75) | 4.00  (0.00, 6.75) | 2.00  (0.00, 6.00) | 5.00  (0.00, 8.00) | 5.00  (0.00, 8.00) | 2.00  (0.00, 6.00) |  |
| Neuroticism score^2^ |  |  |  |  |  |  |  |  |  | 0.279 |
| N-Miss | 633 | 641 | 641 | 394 | 404 | 564 | 207 | 219 | 539 |  |
| Median  (Q1, Q3) | 7.00  (4.00, 9.00) | 7.00  (4.00, 9.00) | 7.00  (4.00, 9.00) | 7.00  (4.00, 9.00) | 7.00  (4.00, 9.00) | 6.50  (3.75, 9.00) | 8.00  (5.00, 10.00) | 8.00  (5.00, 10.00) | 6.00  (3.50, 9.00) |  |
| BMI |  |  |  |  |  |  |  |  |  | 0.852 |
| N-Miss | 3 | 4 | 4 | 2 | 3 | 4 | 0 | 1 | 4 |  |
| Median  (Q1,Q3) | 27.88  (24.87, 31.11) | 27.91  (24.87, 31.14) | 27.91  (24.87, 31.14) | 28.20  (25.01, 31.94) | 28.36  (25.02, 32.06) | 28.16  (25.01, 31.56) | 28.27  (25.03, 31.83) | 28.40  (25.03, 32.15) | 28.18  (25.02, 31.78) |  |

1. Baseline; 2. In-person assessment; 3. Online Mental Health Questionnaire

**Table S7.** Descriptive statistics for individuals included in each definition, Generation Scotland cohort (N = 649). P-values relate to Kruskal-Wallis tests (for numeric variables) and Chi-Square tests (for categorical variables) of between-definition differences.

|  | **1+ switch (N=133)** | **1+ switch Augmentation (N=136)** | **1+ switch Augmentation**  **1+ between-class (N=136)** | **2+ switch (N=23)** | **2+ switch Augmentation (N=29)** | **2+ switch Augmentation**  **1+ between-class (N=81)** | **3+ switch (N=8)** | **3+ switch**  **Augmentation (N=14)** | **3+ switch Augmentation**  **1+ between-class (N=78)** | **p-value** |
| --- | --- | --- | --- | --- | --- | --- | --- | --- | --- | --- |
| Age at survey (years) |  |  |  |  |  |  |  |  |  | 0.963 |
| Median  (Q1, Q3) | 46.00  (36.00, 59.00) | 46.50  (36.00, 59.00) | 46.50  (36.00, 59.00) | 45.00  (35.50, 52.50) | 43.00  (35.00, 53.00) | 43.00  (33.00, 58.00) | 42.00  (39.00, 49.75) | 40.50  (36.25, 51.25) | 43.00  (33.50, 58.75) |  |
| Sex |  |  |  |  |  |  |  |  |  | 0.992 |
| Female | 98 (73.7%) | 100 (73.5%) | 100 (73.5%) | 16 (69.6%) | 19 (65.5%) | 59 (72.8%) | 6 (75.0%) | 9 (64.3%) | 57 (73.1%) |  |
| Male | 35 (26.3%) | 36 (26.5%) | 36 (26.5%) | 7 (30.4%) | 10 (34.5%) | 22 (27.2%) | 2 (25.0%) | 5 (35.7%) | 21 (26.9%) |  |
| SIMD Quintile |  |  |  |  |  |  |  |  |  | 0.988 |
| N-Miss | 10 | 10 | 10 | 2 | 2 | 7 | 0 | 0 | 7 |  |
| Median  (Q1, Q3) | 3.00  (2.00, 4.00) | 3.00  (2.00, 4.00) | 3.00  (2.00, 4.00) | 3.00  (1.00, 4.00) | 3.00  (1.00, 4.00) | 3.00  (2.00, 4.00) | 3.50  (1.00, 4.25) | 2.50  (1.25, 4.00) | 3.00  (2.00, 4.00) |  |
| Educational qualification |  |  |  |  |  |  |  |  |  | 1.000 |
| N-Miss | 5 | 5 | 5 | 1 | 2 | 5 | 0 | 1 | 5 |  |
| Primary | 17 (13.3%) | 17 (13.0%) | 17 (13.0%) | 4 (18.2%) | 4 (14.8%) | 8 (10.5%) | 2 (25.0%) | 2 (15.4%) | 7 (9.6%) |  |
| Lower Secondary | 19 (14.8%) | 19 (14.5%) | 19 (14.5%) | 2 (9.1%) | 2 (7.4%) | 10 (13.2%) | 2 (25.0%) | 2 (15.4%) | 10 (13.7%) |  |
| Upper Secondary | 6 (4.7%) | 6 (4.6%) | 6 (4.6%) | 2 (9.1%) | 2 (7.4%) | 3 (3.9%) | 0 (0.0%) | 0 (0.0%) | 3 (4.1%) |  |
| Post-Secondary | 47 (36.7%) | 49 (37.4%) | 49 (37.4%) | 8 (36.4%) | 12 (44.4%) | 33 (43.4%) | 3 (37.5%) | 7 (53.8%) | 31 (42.5%) |  |
| Degree | 22 (17.2%) | 23 (17.6%) | 23 (17.6%) | 2 (9.1%) | 3 (11.1%) | 12 (15.8%) | 0 (0.0%) | 1 (7.7%) | 12 (16.4%) |  |
| Unknown | 17 (13.3%) | 17 (13.0%) | 17 (13.0%) | 4 (18.2%) | 4 (14.8%) | 10 (13.2%) | 1 (12.5%) | 1 (7.7%) | 10 (13.7%) |  |
| Number of MDD codes in EHRs |  |  |  |  |  |  |  |  |  | 0.990 |
| Median  (Q1, Q3) | 3.00  (2.00, 6.00) | 3.00  (2.00, 5.25) | 3.00  (2.00, 5.25) | 3.00  (2.00, 5.50) | 3.00  (2.00, 5.00) | 3.00  (2.00, 5.00) | 5.50  (2.00, 27.50) | 3.50  (2.00, 5.75) | 3.00  (2.00, 5.00) |  |
| Treatment time (months) |  |  |  |  |  |  |  |  |  | 0.861 |
| Median  (Q1, Q3) | 17.94  (4.99, 48.00) | 18.51  (4.99, 50.00) | 18.51  (4.99, 50.00) | 10.02  (3.50, 27.97) | 20.99  (4.96, 39.03) | 22.01  (4.96, 52.96) | 12.98  (2.77, 27.47) | 23.98  (5.98, 62.02) | 23.51  (4.98, 52.72) |  |
| MDD Polygenic Risk Score |  |  |  |  |  |  |  |  |  | 0.838 |
| N-Miss | 7 | 7 | 7 | 1 | 1 | 3 | 0 | 0 | 3 |  |
| Median  (Q1, Q3) | 0.35  (-0.20, 1.14) | 0.35  (-0.22, 1.14) | 0.35  (-0.22, 1.14) | 0.77  (0.20, 1.44) | 0.70  (-0.17, 1.43) | 0.39  (-0.20, 1.28) | 1.43  (0.03, 1.55) | 1.02  (-0.66, 1.52) | 0.37  (-0.21, 1.24) |  |
| N self-reported depression episodes |  |  |  |  |  |  |  |  |  | 0.941 |
| N-Miss | 15 | 16 | 16 | 3 | 4 | 11 | 0 | 1 | 11 |  |
| Median  (Q1, Q3) | 0.00  (0.00, 1.00) | 0.00  (0.00, 1.00) | 0.00  (0.00, 1.00) | 0.00  (0.00, 1.25) | 0.00  (0.00, 2.00) | 0.00  (0.00, 1.00) | 0.00  (0.00, 0.00) | 0.00  (0.00, 2.00) | 0.00  (0.00, 1.00) |  |
| Self-reported age of onset |  |  |  |  |  |  |  |  |  | 0.220 |
| N-Miss | 81 | 81 | 81 | 15 | 16 | 45 | 7 | 8 | 43 |  |
| Median  (Q1, Q3) | 27.50 (19.75, 37.25) | 27.00  (19.00, 36.50) | 27.00  (19.00, 36.50) | 20.00  (17.50, 25.00) | 21.00  (17.00, 25.00) | 24.00  (17.75, 35.75) | 21.00  (21.00, 21.00) | 21.00  (18.00, 23.25) | 24.00  (18.00, 36.50) |  |
| Mood Disorder Questionnaire total score |  |  |  |  |  |  |  |  |  | 0.831 |
| N-Miss | 70 | 70 | 70 | 14 | 16 | 43 | 5 | 7 | 41 |  |
| Median  (Q1, Q3) | 3.00  (1.00, 7.00) | 3.00  (1.00, 7.00) | 3.00  (1.00, 7.00) | 6.00  (3.00, 10.00) | 6.00  (3.00, 10.00) | 4.50  (1.00, 7.00) | 0.00  (0.00, 5.50) | 6.00  (0.00, 9.00) | 4.00  (1.00, 7.00) |  |
| Destination antidepressant class |  |  |  |  |  |  |  |  |  | 0.234 |
| MAOIs | 0 (0.0%) | 0 (0.0%) | 0 (0.0%) | 0 (0.0%) | 0 (0.0%) | 0 (0.0%) | 0 (0.0%) | 0 (0.0%) | 0 (0.0%) |  |
| Other | 31 (23.3%) | 31 (22.8%) | 31 (22.8%) | 5 (21.7%) | 6 (20.7%) | 27 (33.3%) | 2 (25.0%) | 3 (21.4%) | 27 (34.6%) |  |
| SARIs | 1 (0.8%) | 1 (0.7%) | 1 (0.7%) | 0 (0.0%) | 0 (0.0%) | 1 (1.2%) | 0 (0.0%) | 0 (0.0%) | 1 (1.3%) |  |
| SNRIs | 16 (12.0%) | 17 (12.5%) | 17 (12.5%) | 4 (17.4%) | 5 (17.2%) | 19 (23.5%) | 3 (37.5%) | 4 (28.6%) | 20 (25.6%) |  |
| SSRIs | 82 (61.7%) | 83 (61.0%) | 83 (61.0%) | 14 (60.9%) | 17 (58.6%) | 31 (38.3%) | 3 (37.5%) | 6 (42.9%) | 27 (34.6%) |  |
| TCAs | 3 (2.3%) | 4 (2.9%) | 4 (2.9%) | 0 (0.0%) | 1 (3.4%) | 3 (3.7%) | 0 (0.0%) | 1 (7.1%) | 3 (3.8%) |  |
| General Health Questionnaire total score |  |  |  |  |  |  |  |  |  | 0.077 |
| N-Miss | 7 | 7 | 7 | 0 | 0 | 4 | 0 | 0 | 4 |  |
| Median  (Q1, Q3) | 2.00  (0.00, 9.00) | 2.00  (0.00, 9.00) | 2.00  (0.00, 9.00) | 4.00  (1.50, 10.50) | 6.00  (2.00, 11.00) | 2.00  (0.00, 9.00) | 4.50  (1.00, 10.25) | 7.00  (1.50, 10.75) | 2.00  (0.00, 7.75) |  |
| Neuroticism total score |  |  |  |  |  |  |  |  |  | 0.936 |
| N-Miss | 12 | 12 | 12 | 2 | 3 | 7 | 0 | 1 | 7 |  |
| Median  (Q1, Q3) | 7.00  (5.00, 9.00) | 7.00  (4.75, 9.25) | 7.00  (4.75, 9.25) | 7.00  (5.00, 9.00) | 7.00  (5.25, 9.00) | 7.00  (4.00, 9.00) | 7.50  (5.75, 9.00) | 8.00  (6.00, 9.00) | 7.00  (4.00, 9.00) |  |
| BMI |  |  |  |  |  |  |  |  |  | 0.245 |
| N-Miss | 5 | 5 | 5 | 0 | 0 | 3 | 0 | 0 | 3 |  |
| Median  (Q1, Q3) | 28.20  (24.63, 32.10) | 28.24  (24.64, 32.13) | 28.24  (24.64, 32.13) | 31.93  (24.64, 32.79) | 31.93  (25.75, 34.06) | 28.44  (24.30, 32.79) | 32.66  (29.65, 36.33) | 32.66  (28.04, 36.15) | 28.63  (24.13, 32.79) |  |

**Table S8.** Descriptive statistics for the Treatment Resistant Depression definitions that included ECT in the DataLoch cohort (N = 51,283). Column headings describe the inclusion criteria for each definition, any of which can be met for inclusion. Note descriptive statistics for the 1+ switch, augmentation and ECT definition have been censored due to small differences in sample size with the main analyses, to avoid statistical disclosure.

|  | **1+ switch**  **Augmentation**  **ECT**  **(N~13,643) (27%)** | **2+ switch**  **Augmentation**  **ECT**  **(N=5,506) (11%)** | **3+ switch**  **Augmentation**  **ECT**  **(N=2,295) (4%)** | **p-value** |
| --- | --- | --- | --- | --- |
| Age at end of follow-up (years) |  |  |  | 0.003 |
| Median  (Q1, Q3) | - | 62.00  (54.00, 74.00) | 61.00  (54.00, 72.00) |  |
| Sex |  |  |  | 0.269 |
| Female | - | 4,109 (74.6%) | 1,740 (75.8%) |  |
| Male | - | 1,397 (25.4%) | 555 (24.2%) |  |
| SIMD Quintile |  |  |  | 0.236 |
| Median  (Q1, Q3) | - | 3.00  (2.00, 4.00) | 3.00  (2.00, 4.00) |  |
| Ethnicity |  |  |  | 0.950 |
| Asian | - | 72 (1.3%) | 26 (1.1%) |  |
| Mixed | - | 22 (0.3%) | 11 (0.5%) |  |
| Not Stated | - | 348 (6.8%) | 144 (6.3%) |  |
| Other | - | 61 (1.1%) | 24 (1.0%) |  |
| White | - | 5,003 (90.9%) | 2,090 (91.1%) |  |
| Number of MDD codes in EHRs |  |  |  | <0.001 |
| Median  (Q1, Q3) | - | 4.00  (2.00, 7.00) | 4.00  (2.00, 8.00) |  |
| Treatment time (months) |  |  |  | 0.166 |
| Median  (Q1, Q3) | - | 17.97  (5.98, 45.99) | 18.04  (6.05, 48.00) |  |
| Age of first MDD code (years) |  |  |  | 0.001 |
| Median  (Q1, Q3) | - | 43.00  (35.00, 74.00) | 42.00  (34.00, 53.00) |  |
| Destination antidepressant class |  |  |  | 0.120 |
| MAOIs | - | 24 (0.4%) | 15 (0.7%) |  |
| Other | - | 896 (16.3%) | 344 (15.0%) |  |
| SARIs | - | 466 (8.5%) | 196 (8.5%) |  |
| SNRIs | - | 1,221 (22.2%) | 568 (24.7%) |  |
| SSRIs | - | 1,631 (29.6%) | 664 (28.9%) |  |
| TCAs | - | 1,268 (23.0%) | 508 (22.1%) |  |

**Table S9.** UK Biobank field codes used to identify covariates.

| **Field ID** | **Covariate** |
| --- | --- |
| f.21022 | Age |
| f.31 | Sex |
| f.22189 | Townsend deprivation index |
| f.6138 | Educational qualification |
| f.21000 | Ethnicity |
| f.29000 | Self-report depression diagnosis |
| f.29034 | Self-report age of first diagnosis |
| f.20107, f.20110, f.20111 | Family history of severe depression |
| f.29111 | Self-reported self harm |
| f.2050, f.2060, f.2070, f.2080, f.20507, f.20508, f.20510, f.20511, f.20513, f.20514, f.20517, f.20518, f.20519 | PHQ4 score |
| f.20441, f.20446, f.20441, f.20449, f.20450, f.20439, f.20440 | CIDI severity |
| f.29040, f.29041, f.29042, f.29043, f.29044, f.29045, f.29046 | Any antidepressant helped |
| f.29047, f.29048 | Non-drug treatment helped |
| f.29058, f.29059, f.29060, f.29061, f.29062, f.29063, f.29064 | GAD7 severity |
| f.20127 | Neuroticism score |
| f.21001 | BMI |
